# Realizing the promise of long-acting antiretroviral treatment strategies for individuals with HIV and adherence challenges: an illustrative case series

**DOI:** 10.1186/s12981-022-00477-w

**Published:** 2022-11-26

**Authors:** Christin Kilcrease, Hasiya Yusuf, Joan Park, Aaron Powell, Leon James RN, Jacob Oates RN, Brittany Davis LMSW, Ethel D. Weld, Kelly E. Dooley, Renata Arrington-Sanders, Allison L. Agwu

**Affiliations:** 1 Department of Clinical Pharmacy, Johns Hopkins Hospital, Baltimore, MD USA; 2 Department of Medicine, Division of Infectious Diseases, Johns Hopkins School of Medicine, Baltimore, MD USA; 3 Department of Pediatrics, Division of Pediatric Infectious Diseases, Johns Hopkins School of Medicine, Baltimore, MD USA; 4 Department of Pediatrics, Division of General Pediatrics, Johns Hopkins School of Medicine, Baltimore, MD USA; 5 Department of Medicine, Division of Clinical Pharmacology, Johns Hopkins School of Medicine, Baltimore, MD USA; 6 Department of Medicine, Vanderbilt University Medical Center, Nashville, TN USA; 7 7Department of Pediatrics, Division of Adolescent and Young Adult Medicine, Johns Hopkins School of Medicine, Baltimore, MD USA; 8 Pediatric Adolescent Young Adult HIV/AIDS Program Medical Director, Accessing Care Early (ACE) Clinic, Johns Hopkins University School of Medicine, 21287 Baltimore, MD USA

**Keywords:** Injectable antiretroviral, Nonadherence, Outcomes, HIV/AIDS, Care coordination, Long-acting

## Abstract

**Background:**

Adherence to antiretroviral treatment (ART) remains the cornerstone of optimal HIV outcomes, including viral suppression (VS), immune recovery, and decreased transmission risk. For many people with HIV (PWH), particularly those with early-acquired HIV, structural, behavioral, and cognitive barriers to adherence and competing priorities related to life events may be difficult to overcome, resulting in nonadherence. Long-acting injectable antiretroviral therapies (LAI-ART) may be a useful strategy to overcome some of these barriers. However, to date, the approved LAI-ART strategies (e.g., cabotegravir and rilpivirine (CAB/RPV)) have targeted those who have already attained viral suppression, precluding their use in the 40% of adolescents and young adults (AYA) that VS has eluded.

**Case presentation:**

Ms. X is a 30-year-old woman with perinatally-acquired HIV and barriers to adherence. Despite many interventions, she remained persistently viremic, with resultant immune suppression and multiple comorbid opportunistic conditions, and viral load (VL) > 10,000,000 copies/ml. Given her longstanding history of poor adherence to an oral regimen, a switch to monthly intramuscular (IM) injections and biweekly infusions of ibalizumab were initiated leading to decreased viral load to 8,110 copies/ml within two weeks. Ms. H is a 33-year-old woman with cognitive limitations due to childhood lead poisoning. Her viral load trajectory took a downward turn, precipitated by various life events, remaining elevated despite intensive case management. Initiation of LAI-ART (CAB/RPV) in this patient led to an undetectable VL (< 20 copies/ml) within two months of treatment initiation. Miss Y. is a 37-year-old woman with perinatally-acquired HIV and chronic challenges with nonadherence and longstanding immunosuppression with CD4 < 200 cells/mm^3^ for > 5 years. She received a 1-month oral lead-in (OLI) of cabotegravir/rilpivirine, followed by the injectable loading dose. She has since adhered to all her monthly dosing appointments, sustained VS, and transitioned to a bi-monthly injection schedule.

**Conclusion:**

These three individuals with HIV (perinatally and non-perinatally acquired) with longstanding nonadherence and persistent viremia were successfully initiated on LAI-ART through the process of care coordination and the collective efforts of the care team, highlighting the barriers, challenges, and the multidisciplinary coordination needed to assure successful implementation of this strategy for the most vulnerable of patients.

## Background

Antiretroviral treatment (ART) has transformed HIV infection from an acute life-threatening condition to a chronic manageable one and has reduced HIV/AIDS-associated deaths by nearly 70% since the beginning of the epidemic [1, 2]. ART remains the cornerstone for optimal HIV outcomes, including viral suppression (VS), immune recovery, and decreased transmission risk. Achieving optimal treatment outcomes hinges on treatment adherence [3, 4]. However, many people with HIV (PWH) face structural, logistical, behavioral, and cognitive barriers to adherence [5]. Barriers such as internal and external stigma, low health literacy, low socio-economic status, mental health/substance use disorders, or competing priorities related to life events, caregiving, or unstable housing, may be difficult to overcome, and can result in nonadherence [5, 6]. Nonadherence is further worsened by treatment fatigue imposed by the burden of oral ART use, particularly for people with early acquired HIV (defined as HIV diagnosed before the first decade of life) or who have been on medications all their lives [5].

The recently approved long-acting injectable antiretroviral therapy (LAI-ART) formulations of cabotegravir, an integrase strand transfer inhibitor, and rilpivirine, a non-nucleoside reverse transcriptase inhibitor [7], have shown high efficacy in achieving HIV viral suppression across multiple randomized clinical trials [8, 9]. To minimize burden, considerable efforts have been made to simplify these regimens with monthly or bi-monthly dosing and, recently, making the month-long oral lead-in (OLI) optional [8, 10, 11].

LAI-ART may be a useful strategy to overcome the barriers posed by long-term oral ART. However, viral suppression remains a prerequisite for LAI-ART initiation to date, precluding its use in the 40% of adolescents and young adults (AYA) that VS has eluded [12]. In this case series, we report on three adults with both perinatally- and non-perinatally acquired HIV with longstanding nonadherence and advanced immunosuppression on oral ART who initiated LAI-ART. We highlight the intensive, multidisciplinary care coordination needed to ensure this strategy’s successful implementation and the resultant prompt virologic response in all three cases to the regimen.

The patients described in this report are cared for in a youth-focused multidisciplinary care program, the Accessing Care Early (ACE) Clinic [13]. Embedded within the adult clinic, eligibility for entry into ACE includes age 18–30 years with ≥ 1 criteria: transfer from pediatric care, mental health diagnosis, substance use, or identified adherence barriers. Compared to AYA followed in the standard of care adult program, AYA followed in ACE had higher rates of retention, associated with more frequent social work visits, nurse phone calls, and peer navigator interaction. Youth in ACE did not, however, have significantly higher rates of viral suppression, underscoring the importance of other strategies, including LAI-ART as a mechanism to attain VS. The core care team included providers, nurses, outreach specialist/navigator(s), a clinical pharmacist, and social work case manager, all working synergistically to address needs and barriers to successful implementation. Additionally, the clinical program is a Ryan White-funded program (Parts A, B, D) [14] which pays for salaries for team members and resources for patients (e.g., transportation, food, childcare assistance) as needed to assure successful implementation.

## Case presentation

### Case 1

Ms. X is a 30-year-old African-American female with perinatally-acquired HIV. Her parents were HIV positive, and her mother passed away from complications of AIDS when she was 15 years old. Clinical records from when the patient was 11.8 years old demonstrate persistent fluctuations in her viral load with only brief episodic VS, last at 16.2 years. Combination therapies received in chronological order from the time of ART initiation were (1) stavudine (d4T), efavirenz (EFV), lopinavir/ritonavir (LPV/RTV); (2) atazanavir (ATV), ritonavir (RTV), emtricitabine/tenofovir disoproxil fumarate (FTC/TDF); (3) darunavir (DRV), RTV, FTC/TDF, raltegravir (RAL); (4) ATV, RTV, FTC/TDF, RAL; (5) ATV, RTV, FTC/TDF; (6) ATV, RTV dolutegravir (DTG), FTC/TDF; (7) DTG, FTC/TDF; (8) DTG, FTC/tenofovir alafenamide (TAF); and (9) DTG/RPV, DRV/cobicistat (DRV/c). During the course of treatment, ART regimens were modified based on virologic failure, resistance-associated mutations (RAMs), and for simplification. Her cumulative resistance included: nucleoside reverse transcriptase inhibitor (NRTI) RAMs L210W, T215E/D, K101E, Y181Y/C, and protease inhibitor (PI) RAMs L63P, A71T/A. No INSTI mutations were identified.

**Fig. 1:**
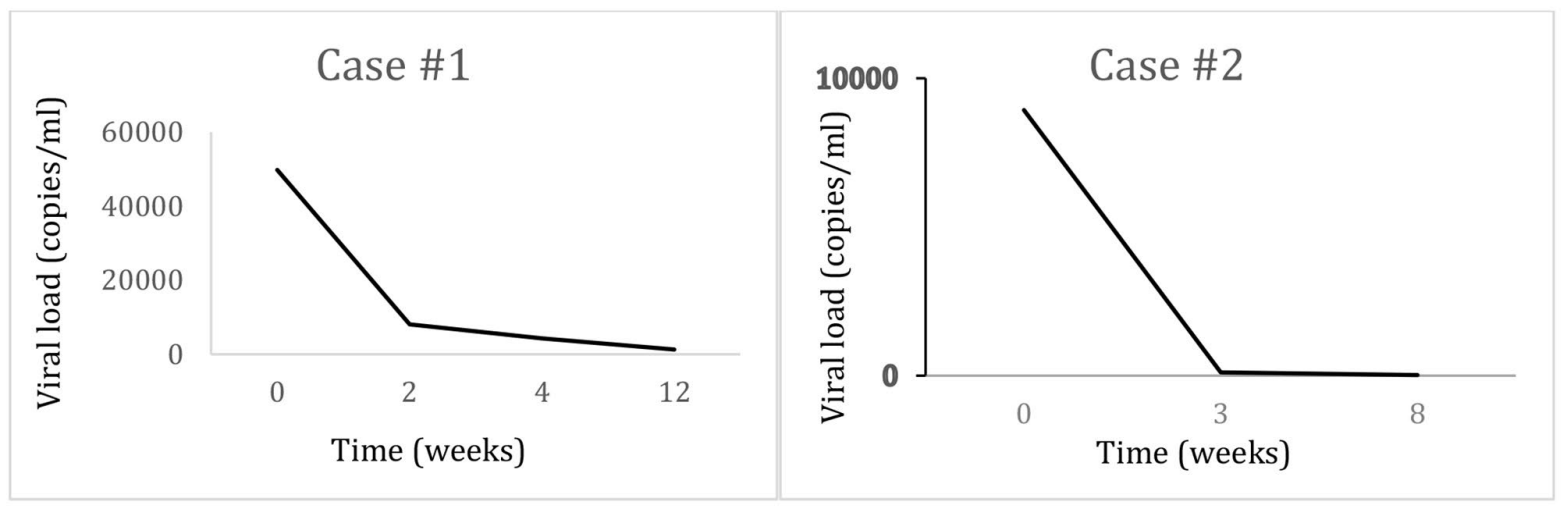
Trends in Viral Loads Following LAI-ART Initiation (Time 0 = initiation of LAI-ART). Case #3 not graphed as VL < 20 at LAI-ART initiation and remained suppressed

Barriers to adherence included cognitive/developmental delay, lack of social support, housing instability, transportation difficulties, poverty, intermittent phone service, lack of childcare, pill burden, medication side effects, and internalized stigma. Various interventions were tried, including simplifying her ART therapy to a once-daily regimen, setting alarm clocks and pillboxes, arranging transportation, assisting with housing, providing adherence counseling, and directly observed therapy (DOT), and connecting her with a patient navigator to help with her outpatient care. Despite these interventions, she remained persistently viremic, even during her two pregnancies, with resultant immune suppression and multiple comorbid opportunistic conditions, including intestinal mycobacterium avium intracellulare (MAI), diffuse molluscum contagiosum, recurrent vaginal infections, and recurrent pneumonia prompting frequent hospitalizations. At 30.6 years of age, she was hospitalized for refractory hypotension in the setting of profuse diarrhea. Her viral load at this time was > 10,000,000 copies/ml. She was re-initiated on her home regimen of DTG/RPV + DRV/c, improving her viral load to 49,800 copies/mL within ten days. Given her longstanding history of poor adherence to an oral regimen, the severe consequences of her advanced immunosuppression, and her proven inability to adhere longitudinally to an oral regimen, the decision was made to switch to monthly intramuscular (IM) injections of CAB/RPV (in clinic), without the OLI, along with biweekly infusions of ibalizumab. Her ibalizumab was initially administered in the clinic and thereafter via home infusion. Her daily oral regimen of DRV/c was continued for additional coverage. Her viral load decreased from 49,800 to 8,110 copies/ml within two weeks. Her most recent viral load was 512 copies/ml four months after initiation of the hybrid regimen (LAI-ART plus oral), her lowest viral load since the age of 16 years ( Fig. 1).

Given her cognitive delay/limitations, care coordination was essential, including engaging the family members in developing the care plan, and assuring that she was available for transportation pick-up. The outreach specialist enabled daily text message medication reminders, appointment reminders, arranged rideshare transportation to the clinic, and assured navigation around the hospital during medication administration days. With this robust support, she has adhered to her injection visit schedule.

The patient’s medical insurance was Medicaid, which provided coverage for direct to inject (DTI) CAB/RPV through the medical benefit and the state-funded copay assistance program, allowing the medication to be initiated promptly and without financial barriers.

### Case 2

Ms. H is a 33-year-old woman with cognitive limitations due to childhood lead poisoning who acquired HIV at 22 years of age when she was sexually assaulted during pregnancy. She initiated LPV/RTV, lamivudine (3TC), and zidovudine (AZT) during pregnancy in 2011 but had to undergo a Cesarean section secondary to rising HIV viral load despite reported medication adherence. She was enrolled in the PROMISE study of ART in pregnancy and assigned to the no-treatment post-partum arm, leading to the temporary discontinuation of ART after delivery. She resumed ART a year later. All combination therapies received in chronological order from the time of ART initiation were (1) LPV/RTV, 3TC, AZT; (2) DRV, RTV, DTG, TDF/FTC; and (3) bictegravir (BIC)/TAF/FTC. During the course of treatment, changes were made to ART regimens based on virologic failure, resistance panels, and for simplification. Her cumulative resistance included: NRTI RAMs M184V and INSTI accessory resistance mutation G163R. Her viral load trajectory took a downward turn, precipitated by various life events, and exacerbated by depression, anxiety, panic attacks, and nausea and vomiting with medications unrelieved by prescribed antiemetics. Her viral load remained elevated despite intensive case management, including DOT and medication reminders. With her persistent viremia (8930 copies/ml), immune decline (CD4 278 cells/mm^3^), and growing concerns that the patient’s continued intermittent adherence to her oral ART would lead to INSTI resistance and preclude her from the use of currently approved LAI-ART, the decision was made to initiate LAI-ART (CAB/RPV) without OLI plus liquid DRV and RTV. Her viral load became undetectable (< 20 copies/ml) within two months of treatment initiation and has remained undetectable since then (Fig. 1). She has adhered to her injection visit schedule.

Critical care coordination included familial involvement, daily treatment reminders, appointment reminders, transportation, and care navigation. She had Medicaid insurance, which provided DTI CAB/RPV coverage through the medical benefit and the state-funded copay assistance program, allowing her to expeditiously begin therapy. The team pharmacist secured prior authorization and financial coverage and developed a therapeutic plan in coordination/conjunction with the medical team.

### Case 3

Miss Y. is a 37-year-old woman with perinatally-acquired HIV, initially diagnosed at five years of age when her mother died of HIV. She has had chronic challenges with nonadherence, which she attributes to limited support systems during her youth as well as currently with competing responsibilities (i.e., employment, childcare), medication side effects (e.g., nausea and grogginess), and internalized stigma and pill aversion when having to take her medication. Her HIV regimen history included (1) monotherapy zidovudine (AZT), (2) nelfinavir, stavudine, and lamivudine (NFV, D4T, 3TC), (3) AZT, abacavir, lamivudine (AZT, ABC, 3TC), (4) atazanavir, ritonavir, tenofovir, emtricitabine (ATV, RTV, TDF/FTC), (5) efavirenz tenofovir, emtricitabine (EFV, TDF/FTC), (6) darunavir, ritonavir, tenofovir, emtricitabine (DRV, RTV, TDF/FTC), and ultimately bictegravir, tenofovir, emtricitabine (BIC/TAF/FTC), all of which she took variably, with persistent intermittent nonadherence. During the course of treatment, ART regimens were modified based on virologic failure, resistance panels, and for simplification. Her cumulative resistance included: NRTI RAMs D67N, T69D, and NNRTI RAMs A98G. No INSTI mutations were identified. Given her nonadherence, she had longstanding immunosuppression with CD4 < 200 cells/mm^3^ for > 5 years with a nadir of 29 cells/mm^3^, with persistent oral mucocutaneous candidiasis and onychomycosis, and various infections, including varicella, pneumonia, and pyelonephritis. Her complaints included decreased energy and appetite with an inability to gain weight. She was first offered LAI-ART in 2019 as part of a study that would require her to adhere to an oral regimen for six months before being randomized to either continue oral ART or to receive LAI-ART; she declined due to a lack of a guarantee that she would receive the LAI-ART. She then continued oral ART, guided by discussions with her provider that LAI-ART would be available clinically by early 2020 if she could attain and maintain a suppressed viral load. Working with her care team, she was transparent about when she was not adherent, and viral loads were not checked at those visits. She was able to suppress viral loads over a 6-month period, and in August 2020, she discontinued her oral ART, received the 1-month OLI of cabotegravir/rilpivirine, followed by the injectable loading dose. She has since adhered to all her monthly dosing appointments, sustained VS, and transitioned to a bi-monthly injection schedule. Regarding care coordination, she received daily medication reminders from the outreach specialist and appointment reminders for adherence to her oral ART regimen and OLI. She had monthly appointments (in-person or telehealth) for check-ins and assessment of adherence with adherence counseling. She too had Medicaid, which provided coverage for LAI CAB/RPV through the medical benefit and the state-funded copay assistance program. The patient was expeditiously started on the regimen as soon as the clinic had an available appointment.

Immune reconstitution inflammatory syndrome (IRIS) was not observed in any of the patients.

## Discussion

This paper reports on three young PWH with poor adherence to oral ART and initial viremia being successfully managed with LAI-ART. Although many clinical trials have reported findings on the efficacy, tolerability, or acceptability of LAI-ART, this is one of the first case series that reports on the outcomes of LAI-ART use among patients facing adherence barriers and unable to viroologically suppress, in a clinical, non-research setting. The three patients presented here have unique circumstances and may not be represented by the experiences reported in the clinical trials. Specifically, the randomized clinical trial of cabotegravir and rilpivirine in treatment-experienced patients (ATLAS study) was limited to PWH who were adherent to ART, were virologically suppressed (VL < 50 copies/ml), and had not experienced virologic failure on oral regimens at least six months prior to study enrollment [15]. These pre-specified criteria inevitably excluded patients with poor adherence to ART and viremia. Likewise, the FDA and insurance providers have set similar criteria for LAI-ART eligibility–patients must be virologically suppressed on the current oral regimen, have VL < 50 copies/ml, and have no reported history of virologic failure [7, 16]. Based on the general perspective that LAI-ART is an effective tool for improving adherence for individuals like those presented in this case series, and considering the high levels of reported adherence and acceptability of the LAI-ART, such limiting criteria potentially exclude those that need the drug the most [17, 18]. These cases also highlight that in some patients, LAI-ART have a potential benefit of reducing pill burden in patients who may still require oral medications.

The need for LAI-ART for patients with adherence barriers to oral regimens is also demonstrated by the results of a recently published compassionate use initiative that allowed providers to request LAI-ART, cabotegravir, and rilpivirine (with or without the month lead-in), for their patients with adherence challenges mostly from psychologic conditions [19]. Notably, 30% of the cohort had perinatally-acquired HIV. Most (63%) of the patients were virologically suppressed at the last follow-up. Of the patients who entered with viremia, 57% achieved VS. Most patients achieved and maintained VS, showing that such patients can benefit from LAI-ART. However, the provision of supportive measures was not described. Successful implementation and execution of LAI-ART for our three patients were made possible through an intensive, multidisciplinary approach involving the coordination of tailored treatment and support services at various levels of patient care. The pharmacist’s role primarily centered around the acquisition of injectable ART. This was done by completing an extensive literature and chart review of the patients’ ART history and labs (e.g., resistance-associated mutations, viral load, etc.), consideration of patient-specific barriers, and lastly, in conjunction with the provider, selection of an optimal ART regimen, which in some cases included other injectable medicines. Overall, this process was expedited because the pharmacist had previously established an institutional workflow for the financial clearance/prior authorization, acquisition, preparation/storage, and patient administration tracking of LAI-ART. Furthermore, a process to establish payment for the medication, through the patient’s insurance coverage and through state-funded assistance programs, was vital for ensuring access to long-acting injectable therapy [14].

Our outreach team contacted patients, ensured closed follow-up through calls and text messaging, provided transportation to get patients with financial or logistical constraints to their appointments promptly, and also facilitated family coordination. Collaboration between providers, laboratory staff, and home care fostered effective viral load monitoring, and funding support through the Ryan White program made the care coordination feasible. Lastly, medical insurance that covered the LAI-ART without significant barriers was key to accessing/securing the medications. The aforementioned collective effort and team coordination resulted in the successful treatment and improved clinical and virologic outcomes for these patients, demonstrating that PWH confronting adherence challenges can and do succeed on LAI-ART when purposive support is provided and strategies and systems of support are in place.

## Data Availability

The datasets used and/or analyzed during the current study are available from the corresponding author on reasonable request.

## List of abbreviations

ART: antiretroviral treatment
VS: viral suppression
PWH: people with HIV
LAI-ART: Long-acting injectable antiretroviral therapies
AYA: adolescents and young adults
VL: viral load
CAB: cabotegravir and
RPV: rilpivirine
OLI: oral lead-in
intramuscular: IM
ACE: Accessing Care Early
d4T: stavudine
EFV: efavirenz
LPV/RTV: lopinavir/ritonavir
ATV: atazanavir
RTV: ritonavir
FTC: emtricitabine
TDF: tenofovir disoproxil fumarate
DRV: darunavir
RAL: raltegravir
DOT: directly observed therapy
DTG: dolutegravir
TAF: tenofovir alafenamide
DRV/c: DRV/cobicistat
RAMs: resistance-associated mutations
NRTI: nucleoside reverse transcriptase inhibitor
3TC: lamivudine
AZT: zidovudine
BIC: bictegravir
DTI: direct to inject
NFV: nelfinavir
ABC: abacavir
ATV: atazanavir
